# Echoes in the Deep: Revealing Influenza A Viruses' Persistence and Microbial Associations in Aquatic Ecosystems

**DOI:** 10.1155/tbed/5586400

**Published:** 2025-10-01

**Authors:** Weijie Chen, Pengfei Yang, Jingjing Hu, Xinyu Liu, Chenyan Jiang, Huanyu Wu, Yuxi Wang, Qingli Yan, Shuiping Lu, Jiasheng Xiong, Xiaoyan Huang, Yue Pan, Fang He, Qi Chen, Siru Hu, Mingquan Chen, Chenglong Xiong

**Affiliations:** ^1^School of Public Health, Fudan University, Key Lab of Public Health Safety, Ministry of Education, Shanghai 200433, China; ^2^Shanghai Changning District Center for Disease Control and Prevention (Shanghai Changning District Health Inspection Institute), Shanghai 200335, China; ^3^Huai'an Center for Disease Control and Prevention, Huai'an 223299, China; ^4^Shanghai Institute of Infectious Disease and Biosecurity, Fudan University, Shanghai 200433, China; ^5^Innostellar Biotherapeutics Co., Ltd., Shanghai 201203, China; ^6^Shanghai Municipal Center for Disease Control and Prevention, Shanghai 200336, China; ^7^Department of Emergency, Huashan Hospital, Fudan University, Shanghai 200040, China

**Keywords:** aquatic ecosystem, H3N2, metagenomics, metatranscriptomics, microbial associations

## Abstract

**Background:**

Influenza A viruses (IAVs) are significant pathogens with complex transmission dynamics in aquatic ecosystems, yet their persistence, evolutionary relationships, and associations with environmental microorganisms remain poorly understood. This study aimed to elucidate the phylogenetic characteristics and ecological associations of IAV in freshwater and seawater ecosystems in Eastern China to inform public health strategies.

**Methods:**

Water samples were collected from three freshwater lakes and a coastal seawater site. Viral particles were concentrated, and nucleic acids were extracted for metatranscriptomic and metagenomic sequencing. Phylogenetic analyses, population dynamics assessments, and microbial association networks were constructed using bioinformatic tools. Statistical tests, including Tajima's *D* and Fu and Li's tests, were applied to evaluate evolutionary trends.

**Results:**

IAV fragments in seawater showed high homology with recent human H3N2 strains from North America (2021–2024), while freshwater-derived fragments aligned with historical avian strains from Asia. Microbial association networks revealed significant associations between IAV and environmental bacteria (e.g., *Brevundimonas aurantiaca*) and fungi (e.g., *Thamnidium*), implying potential ecological associations that may underpin viral persistence. Freshwater environments with higher abundances of Uroviricota exhibited more frequent IAV detection. PERMANOVA confirmed distinct overall microbial community compositions in IAV-positive versus IAV-negative samples across both freshwater and seawater ecosystems (*p* < 0.05).

**Conclusion:**

Aquatic ecosystems, particularly freshwater habitats, may serve as reservoirs for IAV persistence and evolution, driven by complex microbial associations. Regional disparities in viral strain origins highlight the role of migratory waterfowl and environmental transmission routes. Integrated surveillance of aquatic IAV dynamics is critical to anticipate zoonotic risks and mitigate future outbreaks.

## 1. Background

Influenza A viruses (IAVs) are negative-sense, single-stranded, and segmented RNA viruses with a genome consisting of eight segments: PB1, PB2, PA, NP, M, NS, hemagglutinin (HA), and neuraminidase (NA) [1]. Based on the antigenicity of the membrane glycoproteins HA and NA, IAV can be classified into 18 HA subtypes (H1–H18) and 11 NA subtypes (N1–N11), resulting in 198 potential subtype combinations [2]. The distribution and persistence of IAV in the environment are critical factors influencing its transmission among wildlife, livestock, and humans [3]. Many lines of evidence suggest that IAV can survive in water, with its persistence influenced by factors such as temperature, ultraviolet light, pH, chemical agents, and organic matter, as well as variations in survival capabilities among different virus strains [4–6]. Waterfowl are the primary reservoirs of IAV, with most strains affecting mammals originating from avian gene pools [7]. However, current research on IAV in wild bird populations has primarily focused on the birds themselves, with limited investigation into the role of the environment as a source of viral transmission and persistence.

The concept of environmental genomics was first proposed by Schmidt, and later developed into metagenomics by Handelsman, utilizing Illumina technology to study the genomes present in specific environments [8]. This approach has been widely applied in research on microbial diversity. The ocean, being the largest ecosystem on Earth, plays a significant role in nutrient and energy cycling through its microbial populations [9]. However, research on IAV in other aquatic environments, such as rivers, lakes, and wastewater, remains relatively scarce. Therefore, to effectively monitor IAV, understanding the stability of viral particles in feces and environmental water, along with their transmission mechanisms and infection potential, is an important area of investigation.

## 2. Methods

### 2.1. Sample Collection and Virus Particle Concentration

Sampling stations were established in Eastern China, including 15 freshwater sites (five in each of three lakes) and eight seawater sites. At each sampling point, a single water sample comprising 3 L was collected. Sampling was conducted once at each point during the months of January–March 2023 for freshwater sites and January–March 2024 for the seawater site. Water samples were collected 0.5 m below the surface, yielding 3 L per point, filtered through a 0.22 µm membrane, and mixed with AlCl_3_ (final concentration: 20 mg/L) to precipitate viral-like particles. The precipitate was resuspended in ascorbic acid buffer (pH = 6.0) after filtration. DNase I (10 U/mL) and RNase A (1 U/mL) were added to digest exogenous nucleic acids, and the reaction was terminated with 100 mM EDTA and EGTA. The supernatant was collected by centrifugation at 2000 *g* for 5 min and stored at −20°C for subsequent viral DNA extraction.

### 2.2. Nucleic Acid Extraction and Sequencing

For metagenomic analysis, viral DNA was extracted using the MiniBEST Viral DNA Extraction Kit (TaKaRa). For metatranscriptomic analysis, total RNA was extracted using the mirVana RNA Isolation Kit (Thermo Fisher Scientific), following the manufacturer's instructions. Extracted RNA was converted to cDNA using the Maxima First Strand cDNA Synthesis Kit (Thermo Fisher Scientific), with oligo (dT) primers to selectively synthesize cDNA from viral RNA genomes. The resulting cDNA was amplified using the illustra Ready-To-Go GenomiPhi V3 DNA Amplification Kit (GE Healthcare). Metagenomic libraries were constructed using the NEB Next Ultra DNA Library Prep Kit for Illumina (New England Biolabs) and sequenced on the Illumina NovaSeq 6000 platform, yielding 150 bp paired-end files with a depth of approximately 10 Gb.

### 2.3. Contig Assembly and Species Annotation

Sequencing adapters were removed and low-quality nucleotides trimmed using the cutadapt tool (https://cutadapt.readthedocs.io/en/stable/). In the contig assembly process, DNA-matching reads were computationally subtracted from the RNA reads to minimize the influence of DNA contaminants on the metatranscriptomic analysis. Specifically, all unique 30-mers from the DNA libraries were collected, and RNA reads matching these 30-mers were excluded from downstream virus genome assembly using a k-mer based approach. This computational subtraction ensures that the viral contigs assembled from the RNA reads primarily reflect active viral transcription. Unigenes were aligned against bacterial, fungal, archaeal, and viral sequences from the National Center for Biotechnology Information (NCBI) NR database (Version: 2023-03-01) using DIAMOND software (blastp, *e*-value ≤ *e*–5). The LCA algorithm was employed to categorize sequences based on the first branching level, and abundance information was calculated for taxonomic levels.

### 2.4. Similar Sequence Acquisition and Phylogenetic Tree Construction

Strain sequences were obtained from the NCBI. The BLAST function on the NCBI website (https://blast.ncbi.nlm.nih.gov/) was used to include all strains closest to each viral protein gene segment detected in the water. Multiple sequence alignment was performed using the muscle program in MEGA11.0, and the best-fit substitution model was calculated. Phylogenetic trees for various segments from metatranscriptomics and metagenomics were constructed using the neighbor-joining method and 1000 bootstrap replications. To ensure robustness, branches with bootstrap support values < 50% were pruned from the final tree figures, retaining only nodes with ≥ 50% support.

### 2.5. Population Dynamics Analysis

Similar sequence segments obtained from BLAST were summarized by region and sequencing time to explore population dynamics trends. Trends in the detection of different IAV subtypes from metagenomic data were analyzed, and Tajima's *D*, Fu and Li's *D*, and *F*-values were calculated using DnaSP software for detected segments.

### 2.6. Microbial Association Network Construction and Testing

Microbial operational taxonomic units (OTUs) were selected using the QIIME 1.8.0 software. Samples were divided based on the presence or absence of IAV sequences, constructing microbial association networks using OTU abundance data. Only OTUs observed in at least three samples were included for metatranscriptomics, while for metagenomics, OTUs observed in at least half of the samples were considered.

Using abundance data, Spearman correlation coefficients for all possible microbial associations were constructed. Analyses were conducted using the “Hmisc” and “igraph” packages in R. Significant pairwise relationships were used in network construction, visualized using Gephi 0.10.1.

## 3. Results

### 3.1. Detection of IAV

**Figure 1:**
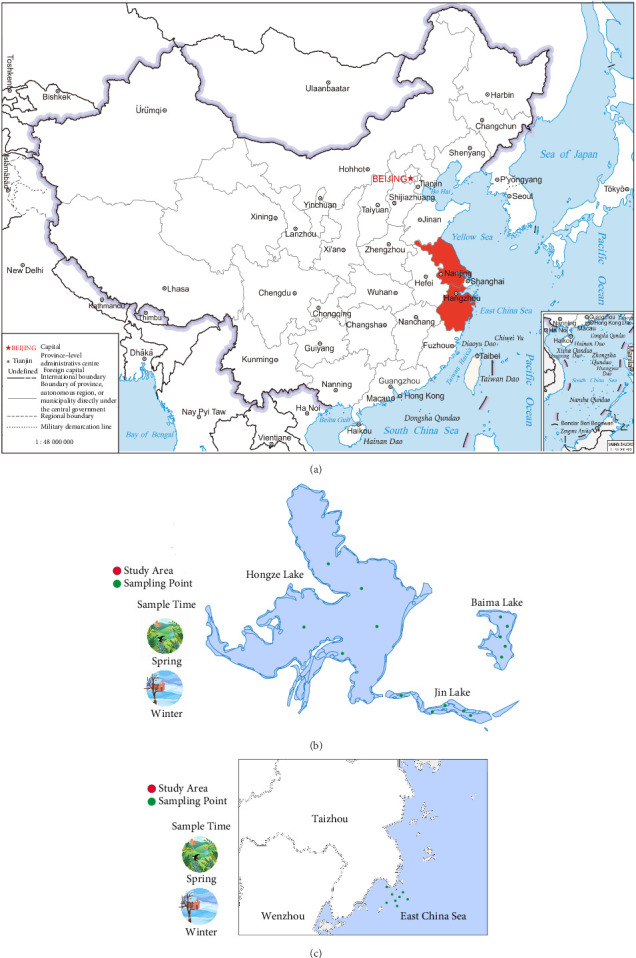
Sampling locations and detection of IAV in freshwater and seawater samples. (a) Map of the study area indicating the collection sites for freshwater samples from Jin Lake, Baima Lake, and Hongze Lake in Huai'an City, Jiangsu Province, and seawater samples from the coastal area of Wenling, Taizhou City, Zhejiang Province. Freshwater samples were collected from January to March 2023, while seawater samples were collected from January to March 2024. (b) Detailed sampling points within Jin Lake, Baima Lake, and Hongze Lake, with five designated sampling locations for each freshwater lake. (c) Sampling points established in the coastal seawater area, showing eight designated locations for seawater sampling.

In this study, the freshwater samples were collected from Jin Lake, Baima Lake, and Hongze Lake in Huai'an City, Jiangsu Province, while the seawater samples were obtained from the coastal area of Wenling, Taizhou City, Zhejiang Province. The sampling period was from January to March 2023 for freshwater and from January to March 2024 for seawater (Figure 1a). Five sampling points were established in each of the three freshwater lakes, and eight sampling points were set up in the seawater area (Figure 1b,c). Through metatranscriptomic sequencing, a total of 14 unigenes were obtained from the seawater samples; in the freshwater samples, IAV was detected only in Hongze Lake, yielding one unigene. Metagenomic sequencing revealed that only one seawater sample contained IAV gene fragments, resulting in two unigenes. Among the freshwater samples, IAV gene fragments were detected in Jinhu and Hongze Lakes, excluding Baima Lake, producing a total of 10 unigenes. The lengths of all unigenes ranged from 150 to 347 bp, and no identical sequences were found between the freshwater lakes or between the freshwater and seawater samples.

### 3.2. Phylogenetic Analysis Fragments

**Figure 2:**
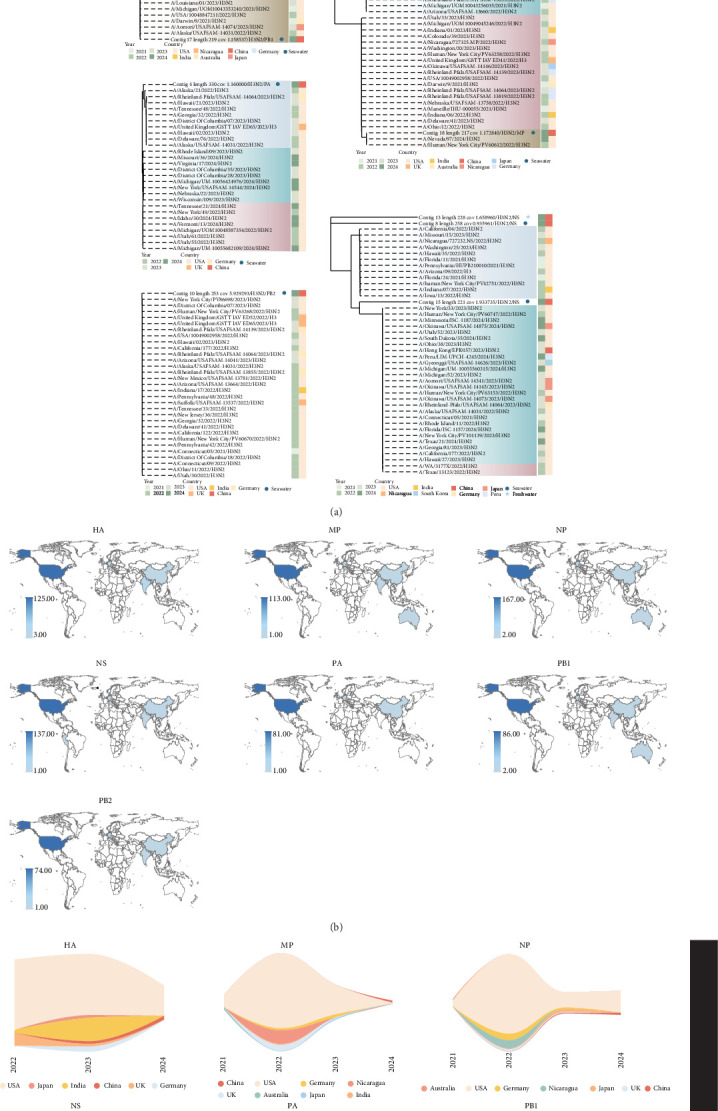
BLAST analysis, phylogenetic relationships and spatiotemporal distribution of IAV fragments identified through metatranscriptomic sequencing. (a) Phylogenetic tree representing the relationships of the HA, MP, NP, NS, PA, PB1, and PB2 gene fragments of IAV identified from aquatic samples. All similar strains obtained from BLAST are H3N2 strains isolated from humans. The phylogenetic tree was constructed using the NJ method with 1000 bootstrap replicates. All branches in this tree have ≥ 50% bootstrap support. The inner color band represents the isolation date of the strains, with different colors indicating different time periods. The outer color band corresponds to the geographic region where the strains were isolated, with each color standing for a specific area. Circles represent IAV fragments isolated from marine water, while stars represent fragments isolated from freshwater. (b) The geographic distribution of IAV strains exhibiting similarity to the seven detected gene fragments. The majority of similar strains are sourced from the United States, with additional contributions from Asia, Australia, and Western Europe. (c) Spatiotemporal trends of similar IAV strains based on isolation date and geographic location.

BLAST comparisons indicated that all detected IAV fragments from the metatranscriptome were highly similar to H3N2 strains circulating over the past 3 years, primarily distributed in North America, Asia, and Europe, with the majority from the United States. Some detections were also noted in Nicaragua, South America, while no related strains have been found in Africa or Australia. Most similar fragments were of human origin (Figure 2a,b). The variation in the number of detected similar fragments allowed us to broadly outline the spatiotemporal changes of the associated subgroups (Figure 2c).

**Figure 3:**
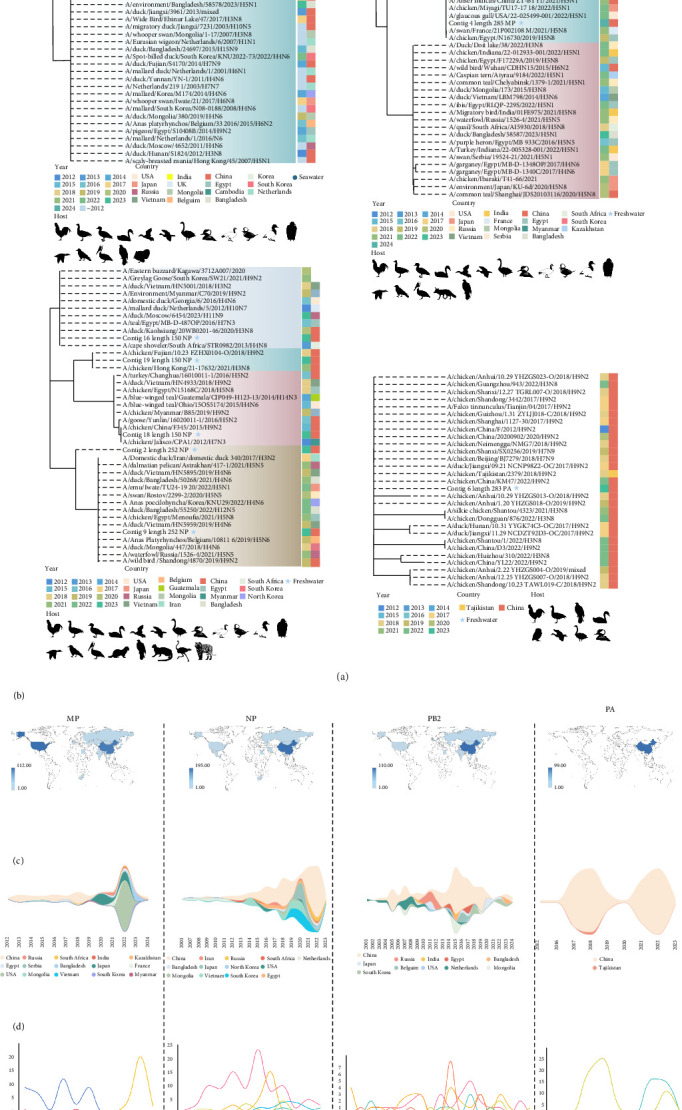
BLAST analysis, phylogenetic relationships and spatiotemporal distribution of IAV fragments identified through metagenomic sequencing. (a) Phylogenetic tree representing the relationships of the MP, NP, PA and PB2 gene fragments of IAV identified from aquatic samples. The phylogenetic tree was constructed using the NJ method with 1000 bootstrap replicates. All branches in this tree have ≥50% bootstrap support. The inner color band represents the isolation date of the strains, with different colors indicating different time periods. The outer color band corresponds to the geographic region where the strains were isolated, with each color standing for a specific area. Circles represent IAV fragments isolated from marine water, while stars represent fragments isolated from freshwater. (b) The geographic distribution of IAV strains exhibiting similarity to the four detected gene fragments. The majority of similar strains primarily originate from Asia, particularly from around China, with a notable emphasis on the PA fragment, where nearly all similar strains are sourced from China. (c) Spatiotemporal trends of similar IAV strains based on isolation date and geographic location. (d) Fluctuation trends of similar fragments across different IAV subtypes. Highlighting the dynamics of strain prevalence over time, the analysis clarifies the dominant subtypes responsible for the expansion and contraction of the IAV population, as depicted in c.

In contrast, all IAV fragments obtained from metagenomic sequencing exhibited a wide temporal range of similar strains, with some strains dating back to the 1960s (Figure 3a). These strains were detected globally, primarily in Asia, Europe, and North America (Figure 3b). Most similar fragments were of avian origin, including those from *Cygnus olor*, *Anser albifrons*, and *Anas clypeata*. Additionally, some strains originated from mammals such as humans, foxes, and tigers. We not only outlined the spatiotemporal changes of the related subgroups (Figure 3c), but also clarified the impact of short-term fluctuations of different subtypes on the dominance of these subgroups (Figure 3d).

To avoid confusion, we validated the dynamic changes of the aforementioned subgroups using Fu and Li's *D* and *F* tests, as well as Tajima's test, with results generally consistent with previous descriptions (Table 1).

### 3.3. Association Network Construction and Features

The significant OTU–OTU relationship network based on species-level abundance derived from the metatranscriptome dataset, which detected IAV fragments, contained 532 nodes and 6203 edges, with 4961 positive associations and only 1242 negative associations. Notably, we observed a significant positive association between *Brevundimonas aurantiaca* and the detection of IAV. In the network, IAV formed a community with environmental bacteria such as *Paraburkholderia xenovorans*, *Clostridioides difficile*, *Pseudomonas japonica*, and *Gammaproteobacteria bacterium*. In the network constructed from the water dataset that did not detect IAV fragments, there were 885 nodes and 31,458 edges, with 31,072 positive associations and only 386 negative associations.

**Figure 4:**
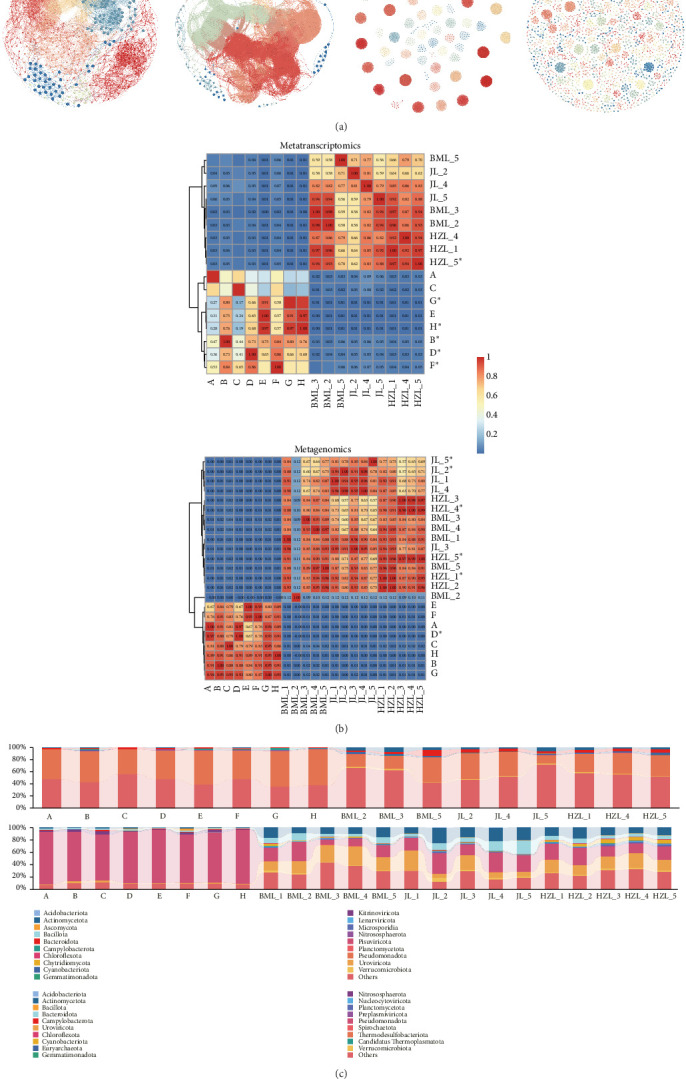
Association networks and microbial composition in water samples with and without IAV detection. (a) Significant OTU–OTU relationship networks based on species-level abundance from metatranscriptomic datasets with (N1) and without (N2) detected IAV fragments, and genus-level abundance from metagenomic datasets with (N3) and without (N4) detected IAV fragments. (b) Sample clustering based on species abundance. Samples marked with an asterisk indicate those in which IAV fragments were detected. Notable differences in microbial structure were observed between freshwater and seawater samples. (c) Analysis of microbial composition differences and variability based on phylum-level abundance. The upper section presents data from the metatranscriptomic analysis, while the lower section displays results from the metagenomic analysis.

The network based on genus-level abundance derived from the metagenomic dataset that detected IAV fragments included 2746 nodes and 99,845 edges, with 59,852 positive associations and 39,993 negative associations. Importantly, we noted significant positive associations between the detection of IAV and genera such as *Lampropedia*, *Candelaria*, *Amanita*, *Polychytrium*, *Thamnidium*, *Jezberella*, *Serpentinimonas*, *Sinimarinibacterium*, and *Aquimonas*, while *Nocardioides* and *Limibaculum* showed significant negative associations. In the network, IAV formed a module with these associated environmental microbes and fungi. The network constructed from the water dataset that did not detect IAV fragments contained 1952 nodes and 4839 edges, with 3762 positive associations and only 1077 negative associations (Figure 4a).

Through sample clustering, we identified significant differences in the microbial composition between seawater and freshwater, with water bodies harboring IAV exhibiting a similar microbial composition pattern (Figure 4b). Through careful observation of microbial community compositions across all samples, we noted distinct patterns in phylum-level abundances. In both metatranscriptomic and metagenomic analyses, Pseudomonadota emerged as the most abundant phylum in both seawater and freshwater samples. Specifically, the abundances of Campylobacterota and Nitrososphaerota were higher in the seawater samples than in the freshwater samples, whereas Verrucomicrobiota, Bacteroidota, Uroviricota, and Actinomycetota were found to be more abundant in the freshwater samples. Similar results were observed in metagenomic analyses, where Pseudomonadota dominated in seawater, while in freshwater, Actinomycetota and Uroviricota also played significant roles alongside Pseudomonadota. Notably, the abundances of Campylobacterota and Nitrosophaerota were considerably lower in the freshwater samples, which exhibited higher levels of other phyla such as Gemmatimonadota, Nucleocytoviricota, and Planctomycetota compared to the seawater (Figure 4c).

**Figure 5:**
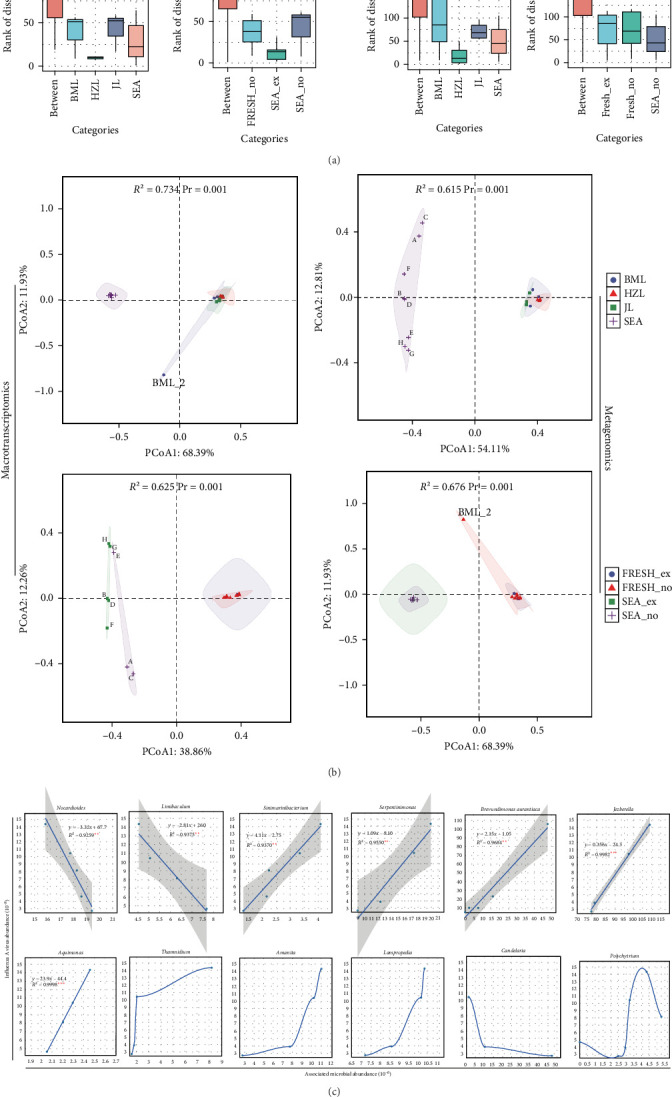
Assessment of microbial community similarity and association dynamics with IAV. (a) Examination of differences in phylum-level microbial composition across various water bodies, categorized by the presence or absence of IAV, using ANOSIM. For water type comparisons (seawater vs. freshwater): *R* values were 0.808 (*p* < 0.001) for metatranscriptomics and 0.674 (*p* < 0.001) for metagenomics. For IAV presence/absence stratification: *R*-values were 0.774 (*p* < 0.001) for metatranscriptomics and 0.516 (*p* < 0.001) for metagenomics. (b) Evaluation of phylum-level microbial community differences among different water bodies, with a focus on the presence or absence of IAV, employing PERMANOVA. For water type comparisons: *R*^2^ values explaining compositional variance were 0.734 (*p* < 0.001) for metatranscriptomics and 0.615 (*p* < 0.001) for metagenomics. For IAV presence/absence stratification: *R*^2^ values were 0.625 (*p* < 0.001) for metatranscriptomics and 0.676 (*p* < 0.001) for metagenomics. (c) Quantification of associations between microbial communities and IAV using appropriate methodologies: linear relationships were analyzed using linear modeling, while nonlinear associations were assessed via locally weighted scatterplot smoothing. A–H represent eight seawater samples, respectively. BML_2 represents Sample 2 from Baima Lake. *⁣*^*∗*^*p* < 0.1, *⁣*^*∗∗*^*p* < 0.05, and *⁣*^*∗∗∗*^*p* < 0.01.

Further supporting our findings, PERMANOVA and ANOSIM analyses confirmed significant differences in microbial composition between seawater and freshwater, with the presence of IAV explaining some of these differences (Figure 5a,b). For microbes that exhibited significant relationships with IAV, we quantified their associations using appropriate linear or nonlinear methods. Our analyses revealed that, beyond clear linear associations (either positive or negative), *Thamnidium* and *Candelaria* displayed notable patterns: they showed positive associations at low concentrations, though these associations plateaued at higher concentrations. Conversely, *Lampropedia* and *Amanita* exhibited opposite trends. Notably, *Polychytrium* displayed a distinct biphasic pattern, with negative associations at both low and high concentrations but a positive association at intermediate concentrations. Collectively, bacterial associations with IAV primarily displayed linear trends, whereas fungal associations were characterized by more complex, nonlinear dynamics (Figure 5c). These observed patterns may reflect potential ecological interactions, though causal mechanisms underlying these associations require rigorous experimental validation to confirm.

## 4. Discussion

In this study, we employed metatranscriptomic and metagenomic sequencing approaches to analyze the presence of IAV and other aquatic microorganisms in the aquatic ecosystems of Eastern China. Our primary aim was to investigate the phylogenetics of IAV strains present in these waters and their potential associations with other microorganisms, thereby contributing to a more comprehensive understanding of IAV epidemiology in aquatic environments and the associated public health risks. By comparing viral sequences, we observed that the IAV fragments identified in the metatranscriptomic dataset (from seawater samples collected in January–March 2024) shared a high degree of homology with human H3N2 strains circulating in North America over the past 3 years [10]. In contrast, IAV fragments detected in the metagenomic dataset (from freshwater samples collected in January–March 2023) were more closely related to historical avian strains from neighboring countries [11]. This spatiotemporal disparity in viral strain characteristics indicates that temporally varying regional IAV prevalence patterns—including local transmission bottlenecks and cross-species spillover events—play a pivotal role in shaping the phylogenetic diversity and persistence trajectories of IAV within China's aquatic systems [12, 13]. Notably, the United States, as a source of these human-like strains, benefits from a robust epidemiological monitoring infrastructure, which likely facilitates the detection of similar viral strains in the environment [14].

The role of water bodies as potential reservoirs for viruses and vectors of viral transmission is significant, particularly in regions with dense populations of migratory waterfowl [15]. Previous research has demonstrated that IAV can persist for extended periods in aquatic sediments, with environmental factors, such as the accumulation of viral particles in aquatic organisms, playing a key role in viral persistence [16]. Studies on filter-feeding organisms, including *Daphnia magna* [17], *Eriocheir sinensis* [18], and *Penaeus japonicus* [19], show that such organisms can accumulate viral particles from contaminated sediments. Given that these organisms are part of the food web, frequently consumed by fish, birds, and even humans, their role in maintaining IAV in the environment and facilitating cross-host transmission should not be overlooked [20].

Furthermore, the phenomenon of microbial symbiosis offers another possible explanation for the prolonged presence of IAV in aquatic environments. Symbiotic relationships, where one microorganism coexists within or on another organism, are widespread in both aquatic and terrestrial ecosystems [21]. These relationships can range from mutualistic to parasitic and are often critical for the survival and ecological success of both the host and the symbiont [22]. For example, mutualistic relationships between aquatic corals and pathogens such as Mycoplasma and Vibrio species are well-documented [23]. Similarly, recent studies, including work by Wang et al. [24] suggest that a coadaptive metabolic relationship between algae and viruses exists, especially during algal blooms. Additionally, the persistence of IAV in aerosols is significantly enhanced when coexisting with pathogens such as *Streptococcus pneumoniae* or *Staphylococcus aureus*, which implies a potential interaction between IAV and other organisms (including microbes, plants, and animals) in aquatic environments [25].

Our results also highlight the importance of microbial diversity in shaping IAV dynamics in aquatic environments. We found a strong association between IAV and certain microbial phyla, including Bacillota, Actinomycetota, Pseudomonadota, Bacteroidota, and fungi such as Mucoromycota and Chytridiomycota, which have previously been associated with both human and environmental habitats [26, 27]. Specifically, we detected microorganisms such as *Limibaculum*, *Aquimonas*, *Sinimarinibacterium*, *Jezberella*, and several fungal species like *Thamnidium*, all of which have previously been identified in association with IAV or related viruses. These findings align with recent studies by Lv et al. [28] which identified a link between the presence of Ascomycota species, including *Candida albicans*, and patients infected with respiratory viruses like H1N1.

An intriguing observation in our study was the differential distribution of IAV fragments across metatranscriptomic and metagenomic datasets. Specifically, IAV fragments were more frequently detected in the metagenomic sequencing from freshwater environments, where a significant presence of Uroviricota was also observed. The positive association between Uroviricota abundance and IAV detection suggests a potential ecological linkage (e.g., copersistence or indirect facilitation) [22]. While horizontal gene transfer mediated by Uroviricota is one plausible mechanism, this remains speculative without evidence of copackaging or chimeric scaffolds. Future studies should validate this hypothesis via Metagenomic scaffolding to detect IAV-Uroviricota hybrid sequences, single-virus genomics or FISH to visualize colocalization, or in vitro models using filter-feeding organisms (e.g., Daphnia) to assess viral coaccumulation [17].

The implications of these findings for public health are considerable. Continued surveillance of IAV in aquatic ecosystems is essential for early detection of potential outbreaks and for monitoring viral mutations and transmission dynamics. Our study did not detect NA gene fragments, likely due to their rapid degradation in aquatic environments and technical constraints in assembling short, variable sequences. Future work should employ subtype-specific enrichment strategies to comprehensively capture all IAV segments. Given that waterfowl populations serve as primary hosts for IAV, their migratory patterns and interactions with the surrounding ecosystems must be closely monitored. Furthermore, the associations between IAV and aquatic microorganisms, especially those within microbial communities, warrant further investigation. These associations may play a crucial role in the replication, evolution, and cross-species transmission of IAV. Understanding these mechanisms will be vital in predicting and mitigating future outbreaks, particularly those that may involve new viral variants with increased transmissibility or resistance to current interventions.

## 5. Conclusions

This study provides new insights into the phylogenetic characteristics, population dynamics, and ecological associations of IAV in China's aquatic environments. The complex interplay between environmental factors, microbial diversity, and viral evolution is central to understanding the epidemiology of IAV in these systems. Our findings suggest that monitoring and controlling IAV in aquatic ecosystems, particularly those inhabited by waterfowl, should be prioritized to mitigate the risks of transmission to humans and other species. Future research should focus on elucidating the specific mechanisms underlying the associations between IAV and aquatic microorganisms, as well as the potential for microbial interventions to control viral persistence in these environments.

## Figures and Tables

**Table 1:** Tajima's *D*, Fu and Li's *D* and *F*-values calculated for different fragments using DnaSP software.

Gene segment	Tajima's *D*	Fu and Li's *D*	Fu and Li's *F*
Metatranscriptomic			
HA	−0.06432	−3.81669*⁣*^*∗∗*^	−2.47233*⁣*^*∗∗*^
MP	0.46413	−2.70388*⁣*^*∗∗*^	−1.82662
PA	−2.06655*⁣*^*∗∗*^	−6.74381*⁣*^*∗∗*^	−5.81291*⁣*^*∗∗*^
PB1	−0.00997	−1.23608	−0.84743
PB2	—	—	—
NS	−0.2375	−5.10110*⁣*^*∗∗*^	−3.37722*⁣*^*∗∗*^
NP	1.02402	−3.23879*⁣*^*∗∗*^	−1.46168
Metagenomic			
MP	0.78153	−1.12535	−0.40221
NP	2.00266*⁣*^*∗*^	−0.73002	0.76549
PA	−1.90085*⁣*^*∗∗*^	−4.62922*⁣*^*∗∗*^	−4.29633*⁣*^*∗∗*^
PB2	0.9375	−4.66157*⁣*^*∗∗*^	−2.71177*⁣*^*∗∗*^

*Note:* In the metatranscriptomic, the PB2 fragment lacks polymorphic sites due to similar sequences, making it impossible to calculate Tajima's *D*, Fu and Li's *D*, and Fu and Li's *F*-values.

*⁣* ^*∗*^ *p* < 0.1.

*⁣* ^*∗∗*^ *p* < 0.05.

## Data Availability

The datasets generated and/or analyzed during the current study are available in the NCBI Sequence Read Archive (SRA) repository, accessible via the persistent link: https://dataview.ncbi.nlm.nih.gov/object/PRJNA1221097?reviewer=v8fv110cijsf6d0nek1sccfdju. Additional metadata and methodological details are provided in Supporting Information 1.

## Supporting Information

Supporting Information Supporting Information 1 provides detailed information about the data deposited in the National Center for Biotechnology Information Sequence Read Archive database, as mentioned in the Data Validity Statement.

The table includes the following columns:

• Accession: The unique accession number assigned to each data entry in the database.
• Study: The study ID that groups related data entries together.
• Object status: Indicates the current status of the data object in the database.
• BioProject accession: The accession number for the BioProject associated with the data.
• BioSample accession: The accession number for the BioSample linked to the data.
• Library ID: The identification number for the specific library.
• Title: A descriptive title for the dataset.
• Library strategy: The sequencing strategy used for the library, such as WGS (Whole Genome Sequencing).
• Library source: Specifies the source of the library, for example, METAGENOMIC.
• Library selection: Describes the method of library selection, such as RANDOM.
• Library layout: Indicates the layout of the library, such as pairedend sequencing.
• Platform: The sequencing platform used, in this case, ILLUMINA.
• Instrument Model: The specific model of the sequencing instrument, like Illumina NovaSeq 6000.
• Design description: A brief description of the experimental design.
• File type: The format of the data files, such as fastq.
• File name: The name of the first file associated with the data entry.
• File name2: The name of the second file (if applicable) associated with the data entry.

This supporting information aims to provide comprehensive details about the data sources and their characteristics, supporting the main manuscript and enabling further verification and analysis by the scientific community.
